# Multivariate Analysis of Linear and Nonlinear Optical Properties in Purine Derivatives: A Predictive Framework from One-Photon Absorption Spectra

**DOI:** 10.1021/acs.jpca.6c02028

**Published:** 2026-06-24

**Authors:** Ian R. Andrade, Leandro H. Zucolotto Cocca

**Affiliations:** Photonics Group, Institute of Physics, Federal University of Goiás, 74690-900 Goiânia, GO, Brazil

## Abstract

The rational design of fluorescent organic molecules is central to the development of advanced linear and nonlinear photonic materials. Purine-based compounds have emerged as promise candidates for several photonics applications due to their structural similarity to biological nucleobases synthetic versatility and favorable photophysical properties. However, their optical characterization typically generates large and complex data sets that are difficult to interpret, particularly when multiple compounds are analyzed simultaneously. Here, we apply principal component analysis (PCA) to a series of purine derivatives to systematically investigate the relationships between molecular descriptors and photophysical performance. The PCA model applied in the optical properties of the set captures 76.8% of the total variance within the first two principal components, enabling clear clustering of molecules according to their electronic structure. Importantly, by applying PCA directly to one- and two-photon absorption spectra, we achieve effective spectral deconvolution with 91.87% and 94.51%, respectively, isolating contributions associated with intensity, spectral shifts, and bandwidth. The robustness of this approach is validated through accurate spectral reconstruction. To extend the analysis toward predictive modeling, multiple linear regression (MLR) was employed to correlate PCA-derived features from one-photon absorption data with the transition dipole moment (*μ_01_*). The proposed PCA-MLR framework effectively captures the intrinsic relationships within the spectra of the studied group, minimizing the need for extensive experimental trials. The resulting model exhibits excellent predictive performance (*R* ^2^ = 0.9728) and accurately estimating the *μ_01_* = 7.07D of an external validation molecule with a deviation of approximately 2.5%. Overall, this PCA-MLR framework provides a powerful and efficient strategy for interpreting complex photophysical data sets and accelerating the design and optimization of organic molecules for linear and nonlinear photonic applications.

## Graphical abstract

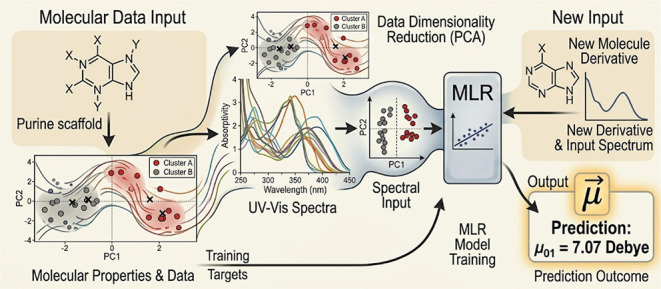

## 1 Introduction

In recent years, the development of advanced photonic materials has been driven by the rational design of fluorescent organic molecules with tailored optical properties.^1–3^ These materials play a central role in applications ranging from fluorescence microscopy,^4^ and optical data storage^5^ to photodynamic therapy^6–8^ and nonlinear optics.^9,10^ In this context, heterocyclic fluorophores have attracted particular attention due to their structural versatility and tunable electronic properties. Among them, purine-based compounds^11,12^ stands out as highly promising systems owing their structural similarity to biological nucleobases,^13^ ease of functionalization,^11^ and their favorable photophysical properties.^14^ These properties include large Stokes shifts,^15,16^ high fluorescence quantum yields,^17^ and the potential for multiphoton absorption processes.^17–19^ Such features make purine derivatives attractive candidates for developing nucleobase analogs and fluorescent probes for biomolecular imaging and spectroscopy.^19–21^

Nitrogen-containing heterocyclic compounds have emerged as highly promising scaffolds by the incorporation of these heteroaromatics into conjugated π-systems, significantly enhancing molecular polarizability and intramolecular charge transfer dynamics.^22–24^ A widely adopted strategy to enhance the optical response of organic chromophores includes the design of push–pull systems^17,25,26^ or increase the conjugation π-conjugation.^27^ The design of push–pull systems can be achieved through introducing electron-donating and electron-accepting substituents at strategic positions^28^ within the molecular scaffold to enhance intramolecular charge transfer.^29,30^ Functionalizing the purine core with appropriate donor–acceptor moieties offers a versatile way to tailor excited-state properties and improve optical responses across a broad spectral range.^31^ In these systems, such modifications significantly influence both linear and nonlinear optical responses. Moreover, these modifications govern critical nonlinear optical parameters, such as the nonlinear refractive index (***n***_2_) and the nonlinear absorption coefficient (**β**), which directly impact the two-photon absorption (2PA) cross-section.^17,22–24,32–34^

2PA is a third-order nonlinear optical process in which a material is excited from the ground state to an excited electronic state^33^ by the simultaneous absorption of two photons. The probability of 2PA is proportional to the square of the incident light intensity.^35^ This relationship results in high spatial selectivity and minimizes photodamage to surrounding regions.^36^ These features have driven the development of organic molecules with enhanced 2PA responses, particularly through systematic molecular design involving π-extension and donor/acceptor substitutions.^37,38^ For example, Mckee et al.^39^ investigated the impact of laser power, scanning speed, hatch distance, and layer height on the structural resolution and fabrication efficiency of microneedles produced via two-photon polymerization. Moreover, Liu and Qian^40^ in their characterization and evaluation of novel BODIPY photosensitizers for singlet oxygen generation via two-photon absorption reported significant 2PA cross-sections evaluated at a single excitation wavelength of 1050 nm, resulting in 661.8 and 715.6 GM for Id-BDPI and Cz-BDPI, respectively, which highlight their potential for two-photon photodynamic therapy applications.

Driven by these promising applications, the linear and nonlinear optical characterization of novel molecules results in a complex and high-dimensional data set, including linear absorption spectra, steady-state and time-resolved fluorescence, solvatochromic behavior, fluorescence anisotropy, and nonlinear absorption responses, usually obtained via techniques like open-aperture Z-scan.^41,42^ While detailed photophysical characterization provides critical insights into the structure–property relationships, the sheer volume and complexity of the data make interpretation challenging. This is especially true when multiple compounds are evaluated simultaneously, especially in the presence of subtle structural variations that lead to correlated optical responses.

In this context, multivariate statistical methods, particularly principal component analysis (PCA),^43^ offer a powerful framework for extracting meaningful information from complex data sets. PCA reduces the dimensionality of correlated variables while preserving the most significant variance in the data set.^44^ By transforming the original data into a set of orthogonal principal components, PCA enables identification of patterns, clustering behavior, and key descriptors governing optical properties. This approach is particularly useful for nonlinear optical studies, where structure–property relationships are influenced by interdependent electronic, geometric, and environmental parameters.^45,46^ It is important to mention that previous studies have successfully applied PCA to analyze the 2PA response of imidazo­[4,5-*b*]­pyridine derivatives (purine isosteres^47^) and donor–acceptor systems.^44^ This allowed for the identification of critical structural descriptors responsible for enhanced cross-section values. Although the photophysics of purine analogues, particularly 2-aminopurine (2AP), one of the most extensively studied representatives, has been thoroughly characterized experimentally,^16,17,48^ the application of PCA to systematically map the linear and nonlinear optical behavior of purine-based chromophores remains largely underexplored.

Here, we bridge this gap by applying PCA to a series of push–pull purine derivatives^16,17^ and already known purines derivatives such as 2AP.^48^ These compounds have been previously characterized in depth using a range of linear and nonlinear spectroscopic techniques.^17^ It should be mentioned that, to the best of our knowledge, the set of compounds investigated in this study encompasses all purine derivatives that have the photophysical properties of interest already reported in the literature. Our main objective is to systematically investigate how molecular and electronic descriptors (such as absorption maxima, emission properties, fluorescence lifetimes, anisotropy, solvatochromism, and 2PA cross-sections) correlate and contribute to the overall photonic performance of these compounds. By integrating experimental data with multivariate statistical analysis, we aim to (i) apply PCA to the experimentally determined photophysical parameters, evaluating the inherent clustering and group differentiation of the purine derivatives based on their optical responses, (ii) perform direct dimensionality reduction on the continuous 1PA and 2PA spectra, effectively decoupling overlapping photophysical features and validating the model through high-fidelity spectral reconstruction, (iii) establish a robust predictive framework, coupling PCA with MLR, capable of accurately forecasting complex optical properties directly from routine linear absorption data, and (iv) demonstrate the usefulness of PCA as an auxiliary tool to support the analysis and interpretation of structure–property relationships in these purine-based fluorophores.

This PCA-guided approach not only complements traditional phenomenological and quantum chemical analyses but also offers a data-driven framework for accelerating the discovery of high-performance fluorophores for applications in fluorescence spectroscopy, nonlinear imaging, and nucleic acid sensing.

## 2 Materials and Methods

### 2.1 Compounds

Purine derivatives are a class of heterocyclic compounds with high thermodynamic stability and notable optical and electronic properties, making them highly relevant for studies in materials physics and biophysics.^49^ The molecular structure, which consists of a fused pyrimidine and an imidazole ring,^50^ imparts a rich π-electron aromatic nature to these compounds. This electronic resonance is responsible for a strong absorption in the ultraviolet (UV–vis) range, with characteristic spectra that are dependent on pH and the local environment.^51^ Furthermore, the presence of functional groups such as amines and carbonyls enables the formation of directional hydrogen bonds, which are crucial for self-assembly into supramolecular structures like the DNA and RNA double helices.^52^ In materials science, purines can act as building blocks for semiconducting polymers and hybrid materials.^53^ Their charge transport properties, influenced by π-orbital overlap, are of particular interest for applications in bioelectronics and optoelectronics.^54^ The interaction of purines with surfaces and nanoparticles is also explored for the development of sensors and detection devices.^55^ A representation of the molecular structures of the 19 molecules studied here is shown in Figure 1.

**Figure 1:**
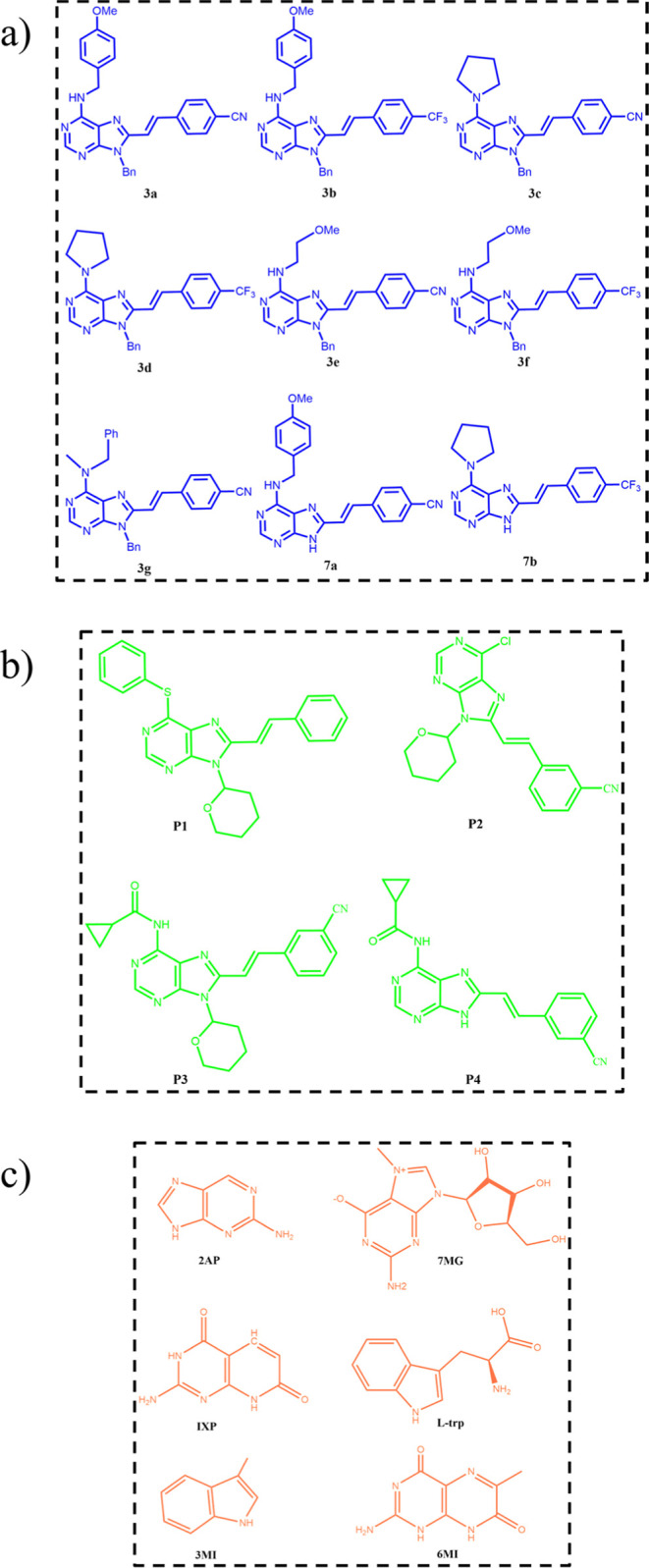
Representation of chemical structures of the investigated purine derivatives. Panels (a) and (b) depict push–pull systems from refs 16 and 17 , respectively, while panel (c) shows additional purine derivatives structures from ref 48.

The multivariate analysis performed herein is based on the comprehensive data set of optical properties previously established by Cocca and co-workers^16,17^ and by Mikhaylov et al.^48^ described in Table 1.

**Table 1:** Optical Parameters of the 22 Molecules Addressed in This Work

Sample	ϕ_*f*_ (%)	Δμ_01_ (*D*)	λ_1*PA*_ (nm)	ε (10^4^ M^–1^ cm^–1^)	σ (GM)	λ_2*PA*_ (nm)
**3a**	75	10.2	370	1.9	20	745
**3b**	81	9.9	357	1.5	19	700
**3c**	62	12.4	391	1.9	24	782
**3d**	79	9.7	375	1.6	24	750
**3e**	63	12.5	370	1.55	20	740
**3f**	65	10.7	357	1.6	18	705
**3g**	63	6.1	383	2.4	30	760
**7a**	81	14.1	373	1.9	19	740
**7b**	99	11.4	367	1.6	28	720
**P1**	8	4	349	3.3	15.3	700
**P2**	14	2.7	334	3.1	11.9	690
**P3**	57	6	340	3.3	23.7	695
**P4**	64	8	340	3.2	22.3	660
**l** **-trp**	12	2.1	279	0.55	0.4	580
**3MI**	2.3	2	280	0.55	0.5	580
**7MG**	1.2	2	258	0.55	3.6	560
**IXP**	7	1	340	1.4	0.7	670
**6MI**		2.8	343	1.009	1.53	701
**2AP-** *H*_2_ ***O***	58	1.1	305	0.556	0.2	612
**2AP-Methanol**	80	1	310	0.631	0.2	622
**2AP-DMSO**	70	1.2	313	0.593	0.4	626
**2AP-Glycerol**	22	1.1	312	0.625	2.4	625

### 2.2 Principal Components Analysis

To systematically analyze the multidimensional data set of spectroscopic parameters, PCA was performed utilizing the Scikit-learn module in Python (v. 3.11). Given that the optical parameters evaluated inherently possess disparate magnitudes and units (e.g., transition dipole moments, spectral bandwidths, and cross-sections), direct application of PCA would mathematically bias the model toward variables with larger numerical scales. To prevent this artifact, each feature was standardized to unit variance according to

1 $$z=\frac{x-\mu }{\sigma }$$

Following standardization in eq 1, where *x* is the raw parameter value, μ is the mean of the feature across all samples, and σ is its standard deviation, PCA was executed via singular value decomposition (SVD) of the covariance matrix to orthogonally transform the correlated variables into a new set of uncorrelated variables, termed principal components (PCs). The optimal number of retained PCs was determined based on the cumulative explained variance, ensuring that the fundamental photophysical information on the data set was captured while effectively filtering out multidimensional noise. The outputs of the PCA were evaluated through two primary projections. Score plots were constructed to project the purine derivatives into the newly defined multidimensional space, facilitating the visual identification of inherent sample clustering, similarities, and potential outliers. Concurrently, loading vectors were extracted to quantify the statistical weight and correlation of each original spectroscopic parameter with the respective principal components, allowing for a physical interpretation of the mathematical axes.

Finally, to rigorously validate the visual groupings observed in the score plots, unsupervised machine learning algorithms were complementarily employed. Both *k*-means clustering and hierarchical clustering were applied directly to the principal component scores, providing a quantitative and objective framework to confirm the structure–property classifications within the data set.

### 2.3 Spectral PCA

To directly evaluate the continuous photophysical profiles, PCA was independently applied to the 1PA and 2PA spectral data. To ensure mathematical validity, the input data matrices were strictly confined to the common intersecting spectral windows shared across all evaluated purine derivatives. In contrast to the preprocessing of discrete parameters, the spectral data were subjected solely to mean-centering, calculated as

2 $$X=x_{i}-\mathrm{x̅}$$

In eq 2, *x*_*i*_ is the original value and x̅ is the mean value of the wavelength across all samples, and *X* is the centered spectra. This step is a methodological choice for continuous spectral data to prevent the artificial amplification of baseline noise in nonabsorbing regions.^56,57^ For both the 1PA and 2PA spectral analyses, scree plots were computed to quantify the cumulative explained variance, specifically validating the high proportion of spectral information retained within the first two principal components. Subsequently, score plots were generated to map the spatial distribution of the molecules in the reduced dimensional space. This projection allowed for the rapid identification of molecular clusters exhibiting analogous overall spectral profiles.

### 2.4 PCA and Multiple Linear Regression (MLR)

To transition from exploratory analysis to predictive modeling, MLR was conducted.^58^ In this step, the principal component scores extracted from the linear absorption spectra were utilized as independent variables to predict target optical parameters. This methodology offers a robust and reproducible framework for applying PCA to spectroscopic data sets, facilitating the understanding of the optical behavior and structure–property relationships across diverse material systems. In this framework, the previously extracted principal component scores functioned directly as the independent predictor variables, while the photophysical parameter of interest served as the dependent response variable. By utilizing these orthogonal scores rather than the highly correlated raw spectral data, the regression strictly avoids multicollinearity artifacts. This predictive relationship is defined by the generalized equation^58^

3 $$y=b_{0}+\sum_{i=1}^{k}b_{i}x_{i}+\epsilon$$

In this equation, *y* is the estimated target parameter, *b*_0_ represents the intercept, *b*_*i*_ denotes the regression coefficients for each *i*-th independent variable (*x*_*i*_), that could be the principal component scores for PCA-MLR, and ϵ accounts for the residual error.

## 3 Results and Discussion

### 3.1 PCA of Photophysical Parameters

For the PCA based on photophysical parameters, a data set containing 22 molecules was analyzed. The feature space was limited to the intersection of parameters available for all compounds, specifically, the linear optical characterization provided by Mikhaylov et al.,^48^ which provides the smallest set of measured variables and defines the maximum number of parameters that could be consistently included in the analysis. Additionally, the fluorescence quantum yield value for the 6MI molecule was not reported in the same study as show in Table 1. To avoid excluding this compound from the data set, the missing value was estimated using the k-nearest neighbors (k-NN) method.^59^ Prior to PCA, all variables were standardized to zero mean and unit variance in order to ensure a proper comparison between parameters with different scales.

For the set of optical parameters evaluated for the purine derivatives, the following variables were considered: fluorescence quantum yield (*ϕ_f_*), permanent dipole moment difference between the first excited state and ground one (*Δμ_01_*), wavelength of 1PA (λ_1PA_), molar absorptivity (ε), the two-photon absorption cross-section (σ), and wavelength of 2PA (λ_2*PA*_), as summarized in Table 1. In addition, the solvent used for each molecule, dichloromethane (DCM), dimethyl sulfoxide (DMSO), or water, was included as the categorical variable. This was implemented using one-hot encoding,^60^ where 1 indicates presence and 0 indicates absence, as shown in Table 2. For the molecules 7MG, IXP, 6MI, and 2AP-glycerol, no solvent descriptor was included since each compound required a distinct solvent. The inclusion of additional unique solvent categories would introduce variables with no shared variance across the data set, leading only to a redistribution of the explained variance among the principal components without providing meaningful correlations.

**Table 2:** Solvent Parameters for Each Molecule with One-Hot Encoding

Amostra	DMSO	DCM	***H***_2_ ***O***
**3a**	0	1	0
**3b**	0	1	0
**3c**	0	1	0
**3d**	0	1	0
**3e**	0	1	0
**3f**	0	1	0
**3g**	0	1	0
**7a**	1	0	0
**7b**	1	0	0
**P1**	1	0	0
**P2**	1	0	0
**P3**	1	0	0
**P4**	1	0	0
**l-trp**	0	0	1
**3MI**	0	0	1
**7MG**	0	0	0
**IXP**	0	0	0
**6MI**	0	0	0
**2AP-** *H*_2_ ***O***	0	0	1
**2AP-Methanol**	0	0	0
**2AP-DMSO**	1	0	0
**2AP-Glycerol**	0	0	0

Using the optical parameters listed in Table 1 and the solvent descriptors in Table 2, the first two principal components (PC1 and PC2) account for 76.82% of the total variance. The cumulative variance explained by the first nine principal components is presented in Figure 2. The high contributions of PC1 and PC2 indicate that the dimensionality of the problem can be significantly reduced without substantial loss of information, enabling a reliable interpretation of the correlations among the optical parameters.

**Figure 2:**
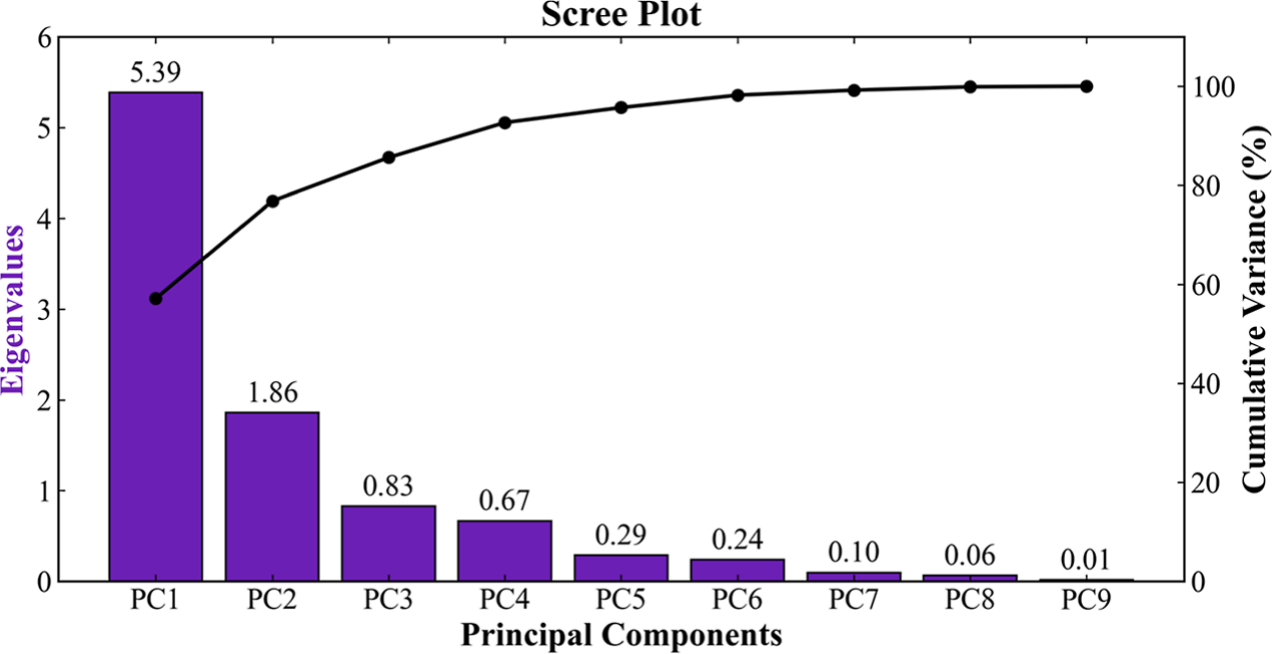
Scree plot showing the eigenvalues and cumulative explained variance from the PCA applied to the optical parameters of the purine derivatives. The first two PCs account for 76.82% of the total variance.

The analysis of PCA scores for the optical parameters showed a clear separation and clustering in 2 different groups, as exhibited in Figure 3. The first cluster (highlighted in red) exclusively comprises the molecules featuring a push–pull architecture, characterized by a unified donor–π–acceptor (D–π–A) conjugated system. Conversely, the second cluster (colored gray) encompasses the well-established, commercially available purine derivatives. Unlike the first group, this subset lacks a common D–π–A structural motif, representing a broader class of standard purine architectures. Both clusters were closed by 90% confidence ellipses through the centroid of each set.

**Figure 3:**
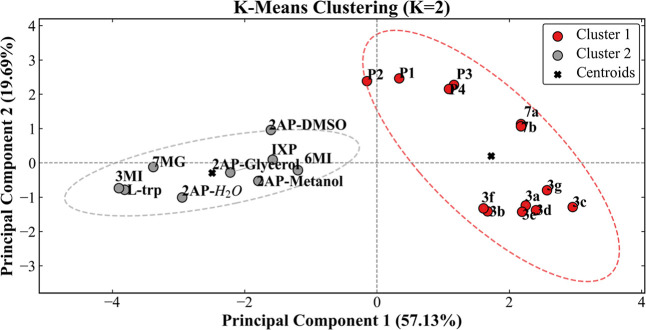
Score plots of the purine derivatives. Each data point represents an individual molecule, and the axes correspond to the principal components that capture the strongest and weakest correlations among their photophysical properties.

The PCA results clearly reveal the clustering behavior of the investigated molecules. PC1 is strongly influenced by the optical parameters, including ϕ_*f*_, μ_01_, λ_1*PA*_, ε, σ, λ_2*PA*_. The strong contribution of these variables allows a clear separation of the data points, reducing the likelihood of artificial clustering in the score plot.

Figure 4 shows the loading plot, which is essential for identifying the variables that contribute most to the variance explained by the principal components. The projection of the variables in the PC1-PC2 space provides information about the direction and magnitude of each spectroscopic parameter. This allows for the identification of the optical properties that most strongly drive the data set distribution, particularly when evaluated in conjunction with the corresponding molecular positions in the score plot. In this representation, each vector starting from the origin corresponds to a variable, and its length is proportional to its contribution to the principal components. The angle between vectors reflects the correlation between variables: small angles indicate strong positive correlation, angles close to 180° indicate negative correlation, and angles near 90° indicate weak or negligible correlation.^61^ The combined interpretation of the score plot (Figure 3) and the loading plot (Figure 4) makes it possible to identify molecular clusters associated with enhanced linear and nonlinear optical responses, according to the positive or negative correlations between the variables and the principal components.

**Figure 4:**
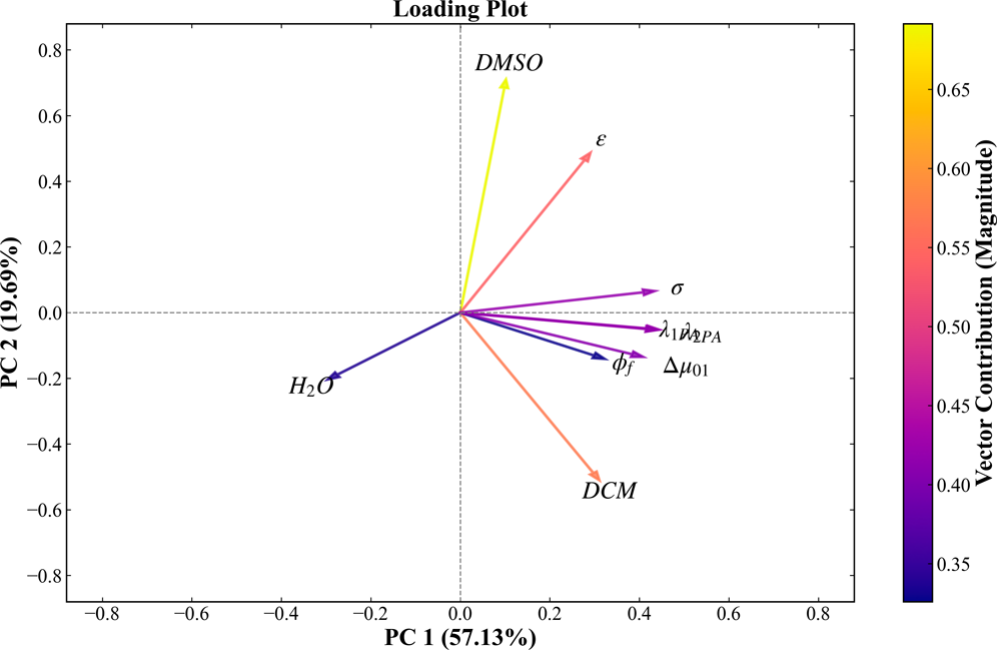
Loading plot of push–pull purines derivatives showing the direction and magnitude of each spectroscopic parameter, where the solvents have pronounced magnitude and the molar absorptivity.

For example, the vector associated with *ϕ_f_* is predominantly oriented in the positive direction of PC1, indicating that PC1 is highly correlated with this optical property. This behavior is consistent with the score plot (Figure 3), where molecules located along the positive PC1 axis correspond to those exhibiting higher *ϕ_f_*. Thus, the loading plot enables a direct correlation between the position of each molecule in the score space and its corresponding photophysical properties. This combined interpretation provides a quantitative framework for understanding the structure–property relationships governing the optical behavior of the purine derivatives. The observed correlations between molecular features and optical responses further highlight the effectiveness of PCA as a tool for linking molecular architecture to optical properties in organic photonic materials.

### 3.2 1PA and 2PA Spectral PCA

While PCA based on scalar photophysical parameters provides valuable classifications regarding solvent effects and other molecular properties, it does not capture the full spectral response of each compound. To address this limitation, the analysis was extended to the linear and nonlinear absorption data by treating the entire spectra as data sets. Applying PCA directly to the UV–vis (1PA) and 2PA cross-section spectra of the selected compounds enables a more comprehensive description of their optical behavior.

Performing a direct PCA on these spectra requires a common wavelength range across all molecules. However, for the full set of 22 compounds (Figure 1), the absorption bands span significantly different regions, restricting the shared spectral overlap to a narrow window of approximately 60 nm (270–330 nm). Conducting PCA over such a limited range would yield insufficient variance representation, failing to capture the primary spectral features and the overall variability of the absorption bands. To overcome this, the data set was restricted exclusively to the push–pull purine derivatives (Figures 1a,b), ensuring a broad, common spectral window for all analyzed samples. This targeted selection enabled the inclusion of the lowest-energy absorption band for each compound, covering 300–450 nm for the 1PA spectra and 600–900 nm for the 2PA spectra. As all variables correspond to intensity values in identical units, the spectral data were mean-centered prior to analysis.

According to the preset established, the first two principal components accounted for approximately 91.87% of the total spectral variance in 1PA data set and 94.51% for 2PA data set (scree and score plots for the spectral analyses are provided in the Supporting Information).

The loading plot captures and identifies wavelengths that contribute most significantly to spectral variance. In the 2PA analysis, these wavelengths corresponded to the same transition as the 1PA;^17,62,63^ consequently, the PCA applied to the 1PA and 2PA spectra is expected to exhibit similar spectral patterns. Notably, molecules with pronounced intramolecular charge transfer (ICT) character show strong loadings at longer wavelengths,^64^ confirming that PCA capture not only intensity-based differences but also spectral shape and position.

The 1PA PC1 loadings in Figure 5a reveal that the first principal component is primarily driven by the spectral position of the absorption band and intensity, alone accounting for 77.93% of the total data set variance. PC2 and PC3 capture spectral shifts and band broadening that result from the experimental response of molecules absorbing at different wavelengths and exhibiting different absorptivity intensities.^65^ This grouping arises from the structural similarities and charge-transfer properties shared by the molecules, despite their distinct linked groups.

**Figure 5:**
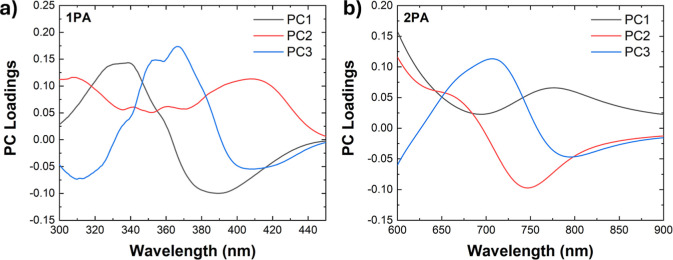
Loading plot showing the first 3PCs of (a) one-photon absorption and (b) two-photon absorption spectra of 13 purines.

Likewise analogous features are observed in the 2PA loadings Figure 5b. In this case, PC1 comprises strictly positive loadings that reflect the overall absorption intensity and mean spectral profile. Furthermore, the subsequent components isolate specific photophysical alterations: PC2 accounts for bathochromic or hypsochromic spectral shifts,^66^ whereas PC3 captures changes in bandwidth and spectral broadening.^65^ The 2PA behavior is primarily governed by the π-conjugation of the central pyridine rings, which serve as the core of the molecular structure^67^ and can impact in the PC2 directly.

The application of PCA to the 1PA and 2PA spectra provides robust evidence for the noncentrosymmetric push-pull architecture, a characteristic clearly reflected in both the experimental data sets and the individual PC loadings. The high degree of correlation between the experimental data and the theoretical model, as indicated by PCA, suggests a high level of confidence in the results. To validate the robustness of the PCA model, the original spectral data were reconstructed utilizing the derived principal component loadings and their corresponding scores, as shown in Figure 6. Notably, the root-mean-square error (RMSE) associated with each presented reconstruction is low, relative to the absolute scale of the spectral intensity. This minimal residual confirms the high fidelity of dimensionality reduction. The complete set of reconstructed spectra can be found in the Supporting Information.

**Figure 6:**
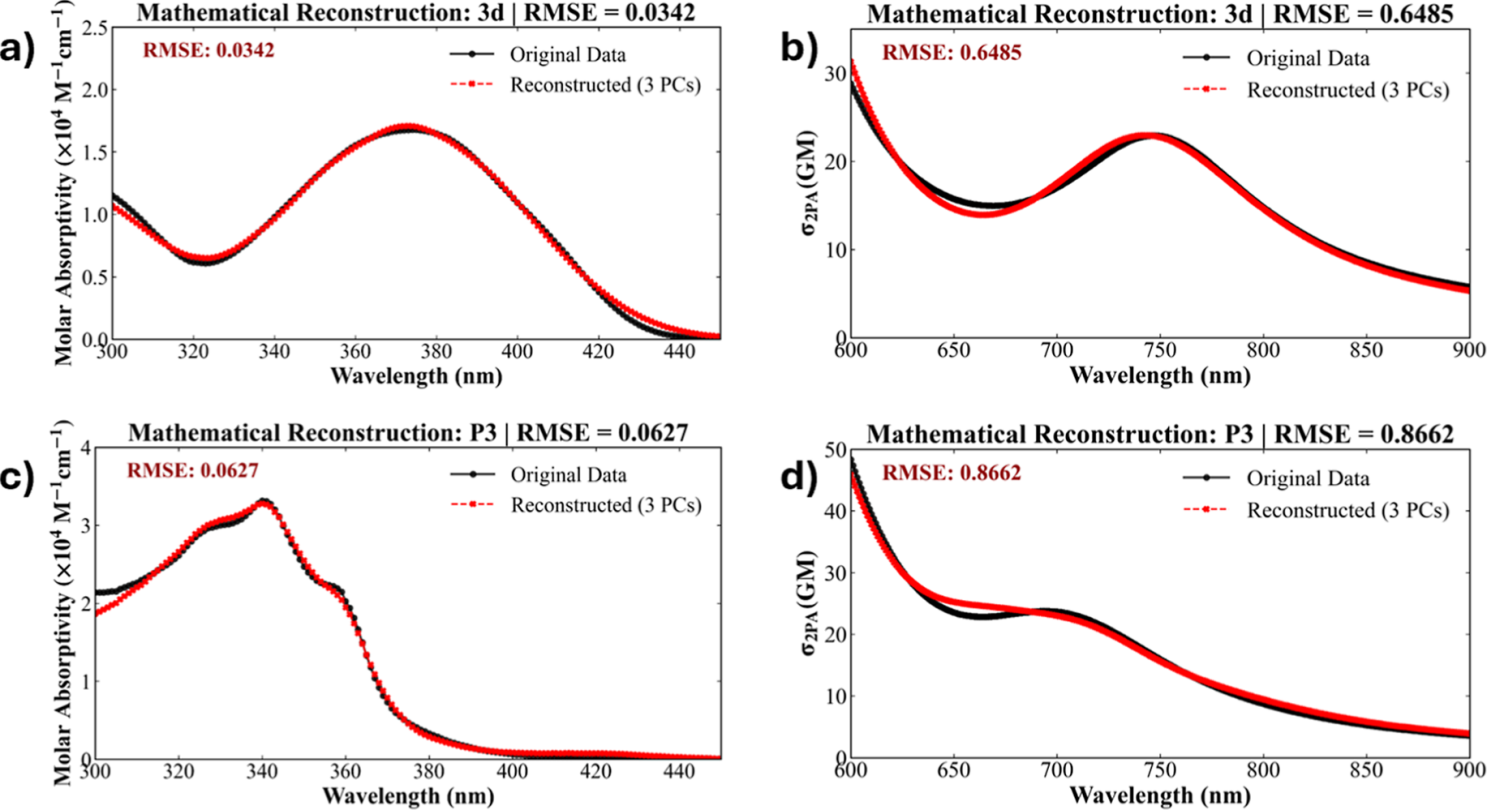
Spectral reconstruction of 1PA (a,c) and 2PA (b,d) profiles based on three principal components. The graphs (a,b) are related with molecule 3d from Figure 1a. The graphs (c,d) are related with molecule P3 from Figure 1b.

### 3.3 PCA-MLR Prediction

Having established that the PCA-based reconstruction reproduces the experimental spectra with minimal residual error, the extracted principal components can be considered to retain the essential photophysical information on the data set. Building on this validated dimensionality reduction, the analysis was extended to the predictive modeling stage. In this way, here we investigate whether key spectroscopic parameters can be estimated using only 1PA spectral data as input variables. This approach is intended as a complementary tool for experimental research, enabling the prediction of optical properties from the standard linear measurements. Such a strategy allows rapid in silico screening of optical responses, reducing the need for exhaustive experimental characterization and providing a practical route to accelerating the rational design and optimization of organic materials for photonic applications.

To perform the predictive modeling, the *μ_01_* was selected as the target physical parameter due to its fundamental role in governing the transition probability and its direct correlation with the optical absorption profile.^68^ A multiple linear regression approach^69^ was employed to stablish a relationship between spectral data and this photophysical property. To rigorously validate the predictive capability of the model, a target molecule (P3 shown in Figure 1b) was intentionally excluded from the data set of 13 push–pull purine derivatives. The PCA was then performed strictly on the remaining 12 molecules, ensuring that the multidimensional feature space was constructed without prior information from the P3 spectrum. Following the dimensionality reduction, MLR was conducted by regressing the experimental μ_01_ values of the 12 molecules training set against their respective principal component scores, yielding a predictive equation of the general form

4 $${\mathrm{\mu ̂}}_{01}=b_{0}+\sum_{i=1}^{k}b_{i}{PC}_{i}$$

In this equation, ${\mathrm{\mu ̂}}_{01}$ is the estimated transition dipole moment, b_0_ is the intercept, b_*i*_ is the regression coefficient, and PC_*i*_ denotes the respective principal component scores. Finally, to test the model, the absorption spectrum of the withheld molecule (P3) was projected onto the established PCA space to extract its scores, which were then applied to the MLR equation to estimate its μ_01_ value.

Applying the coupled PCA-MLR methodology to the 12-purine training set yielded the following predictive equation based on the first three principal components

5 $${\mathrm{\mu ̂}}_{01}=0.1013\cdot {PC}_{1}+0.1927\cdot {PC}_{2}+0.0082\cdot {PC}_{3}+5.5500$$

The regression model exhibited strong predictive linearity, this is evidenced by a coefficient of determination *R* ^2^ = 0.9728, indicating an excellent correlation between the reduced spectral features and the target physical property. To evaluate the predictive capability of the model, the experimental spectrum of the molecule P3 was projected onto the established PCA space. The model estimated a transition dipole moment of ${\mathrm{\mu ̂}}_{01}=7.07D$ . When compared to the reference value of *μ_01_* = 6.9D previously reported by Cocca et al.,^16^ this estimation yields a remarkably low relative error of approximately 2.46%. This high degree of accuracy underscores the robustness of the proposed framework, demonstrating its efficacy as a rapid, concise, and highly auxiliary tool for accelerating the extraction of complex photophysical parameters from standard optical analyses.

Overall, the obtained results confirm the reliability of the PCA-based framework for describing and predicting the optical behavior of purine derivatives, as discussed in the following conclusions.

## 4 Conclusions

In conclusion, this study successfully establishes a robust multivariate statistical framework to comprehensively evaluate and predict the photophysical behavior of purine derivatives. Initially, the application of PCA to established photophysical parameters demonstrated the method’s capability to cluster molecules based on structure–property relationships, despite dimensionality constraints inherent to combined literature data sets. By refining the analytical scope to a consistent spectral window, we performed direct dimensionality reduction on the one- and two-photon absorption spectra of 13 purines set. The extracted principal components effectively decoupled critical, overlapping photophysical features, namely, the overall absorption intensity, spectral shifts, and band broadening. The high fidelity of this mathematical reduction was corroborated by the successful reconstruction of the experimental spectra with minimal residual variance.

Based on this validated model, we advanced the exploratory analysis into a highly accurate predictive tool. By coupling PCA with multiple linear regression, we demonstrated that optical parameters, such as the transition dipole moment, can be reliably forecasted relying exclusively on routine 1PA spectral scores. The external validation yielded an excellent predictive linearity of R^2^ = 0.9728 and a remarkably low relative error ∼ 2.5%. Ultimately, this PCA-MLR methodology provides a powerful auxiliary tool for experimental results, in silico screening strategy that accelerates the determination of optical properties from standard linear measurements, optimizing the rational design of new materials for nonlinear photonics.

## Supplementary Material



## Data Availability

All data sets generated or analyzed during this study are included in this article.

## Notes

PCA applied to the one-photon absorption spectra, including the corresponding scree plot, score plot, and the complete set of reconstructed spectra with their respective root-mean-square error values; and analogous analysis for the two-photon absorption spectra, comprising the scree plot, score plot, and the full spectral reconstruction data set with RMSE values (PDF)
