# Supplementary material for: Multivariate Analysis of Linear and Nonlinear Optical Properties in Purine Derivatives: A Predictive Framework from One-Photon Absorption Spectra
Source: J Phys Chem A. 2026 Jun 24;130(27):5205–14. doi: 10.1021/acs.jpca.6c02028 (PMC13359372; doi:10.1021/acs.jpca.6c02028)
Supplement: Supplementary file 1 [file jp6c02028_si_001.pdf]

Multivariate Analysis of Linear and Nonlinear Optical Properties in Purine Derivatives: A Predictive Framework from One Photon Absorption Spectra

Supplementary Information

Ian R. Andrade[a], and Leandro H. Zucolotto Cocca*[a]

[a] Photonics Group, Institute of Physics, Federal University of Goiás, 74690-900, Goiânia, GO, Brazil

* Authors to whom correspondence should be addressed: leandro.zucolotto@ufg.br and ian_ribeiro@discente.ufg.br

1. PCA for the one-photon absorption (1PA) spectra

The scree plot, show explained variance for each component is illustrated in **Figure SI1**. The figure demonstrates that 91.87% of explained variance with two principal components.

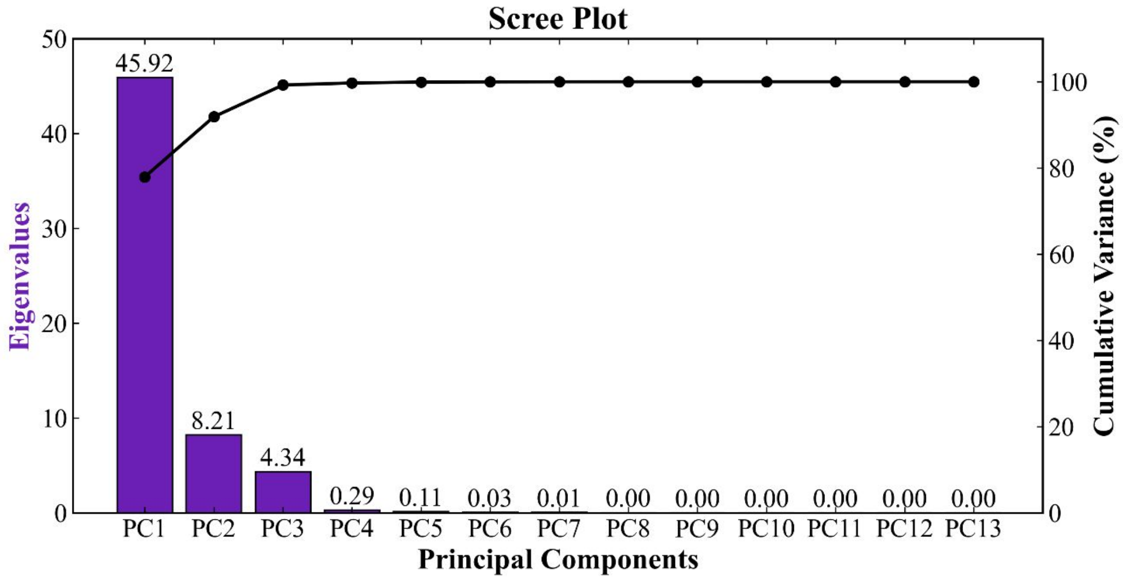

Figure S1. Scree plot for the PCA of 1PA.

The dominant variance contributions of PC1 and PC2 indicate that the spectral data can be rigorously reduced to a two-dimensional subspace with minimal information loss.

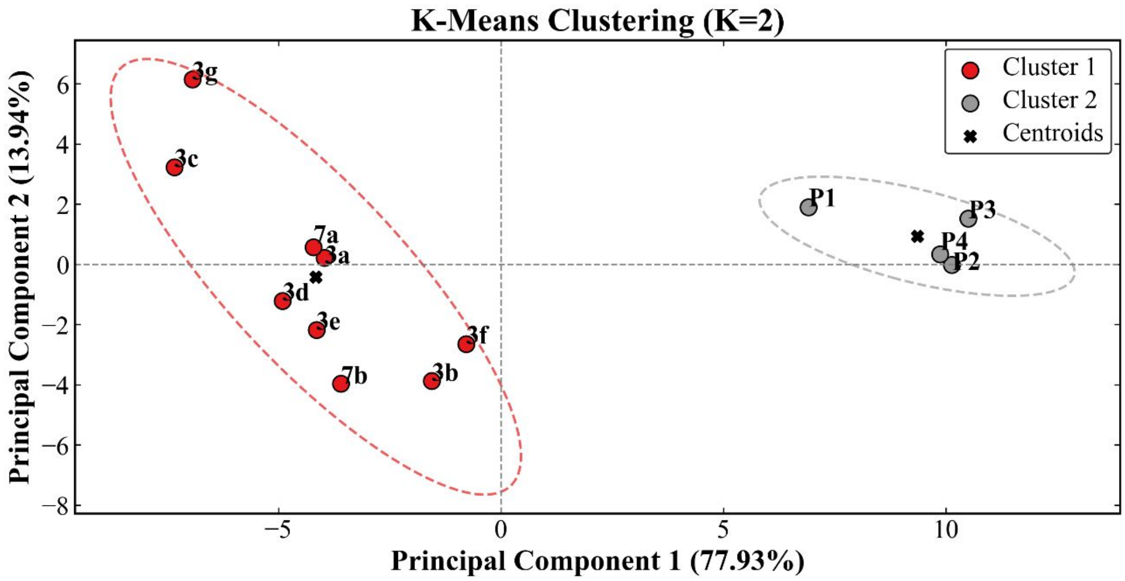

Figure S2. Score plot for the PCA of 1PA.

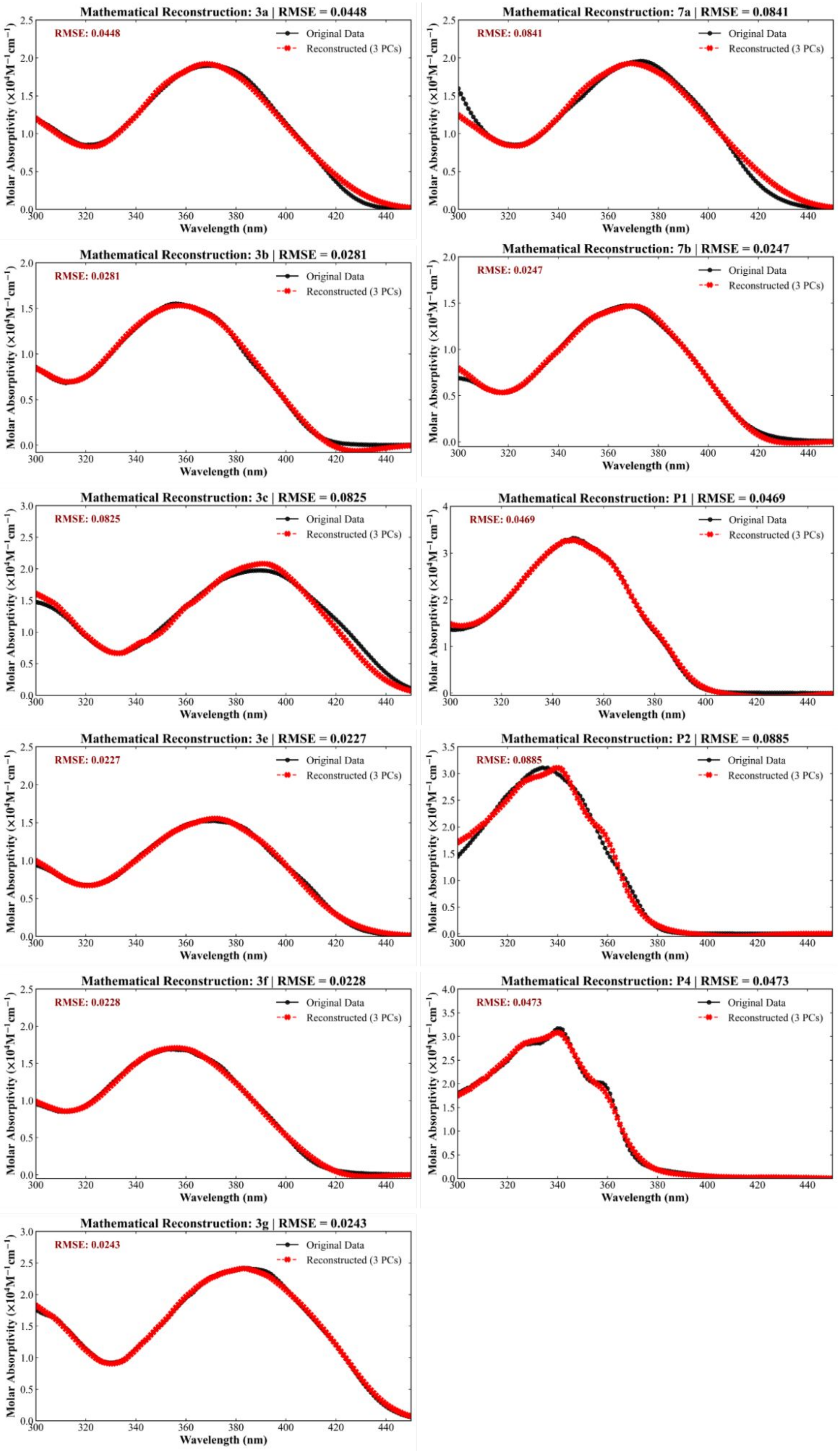

Figure S3. Reconstruction for original spectra used in PCA of 1PA. The reconstructed spectra of 3d and P3 are presented and discussed in the main manuscript.

The score plot for the 1PA specrtra showed a clear separation and clustering in 2 different groups as exhibited in **Figure SI2,** which may indicate different absorption regions due to differences in clustering, and **Figure SI3,** shows all spectral reconstruction and their respective RMSE.

2. PCA for the two-photon absorption (2PA) spectra

For the PAC of 2PA, the **Figure SI4** ilustrate their cumulative variance for each component. The figure show that 94.51% of explained variance with two principal components.

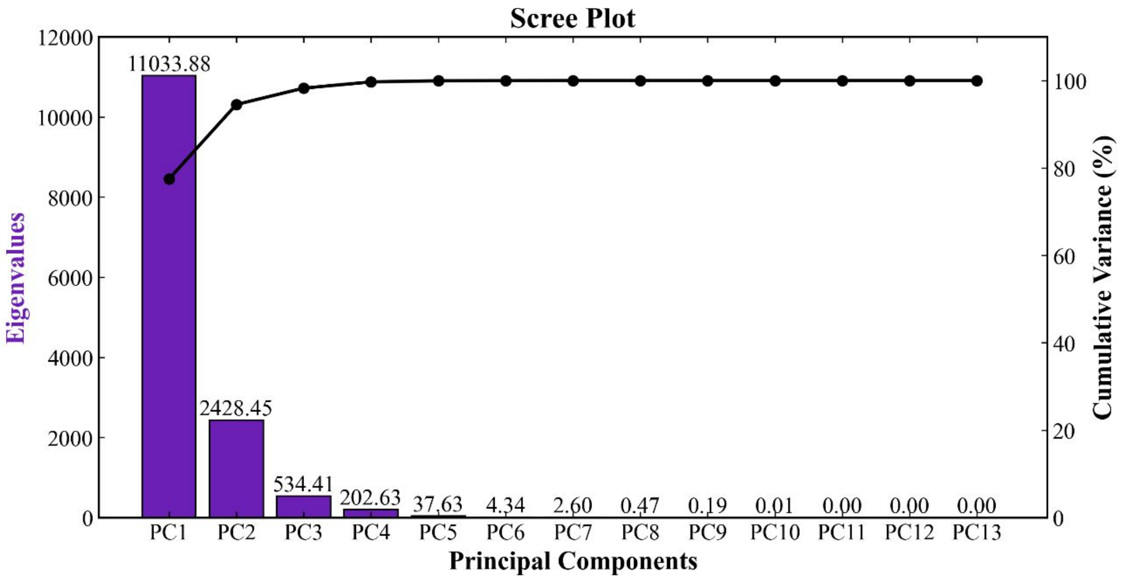

Figure S4. Scree plot for the PCA of 2PA.

The same dominant variance contributions of PC1 and PC2 as to 1PA indicate that the spectral data can be rigorously reduced to a two-dimensional subspace with minimal information loss.

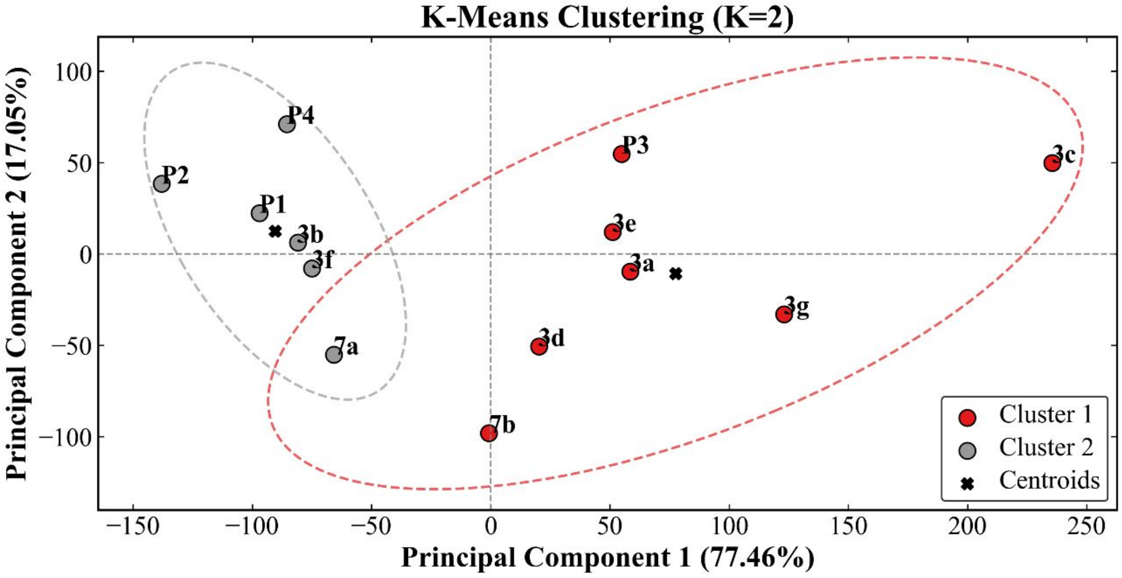

Figure S5. Score plot for the 2PA-PCA.

The analysis of the 2PA spectral score plot in **Figure SI5** reveals a distinct separation of the dataset into two primary clusters, there is a noticeable spatial overlap between them relative to the highly resolved 1PA projection. This increased blending in the multidimensional space reflects a higher intrinsic similarity in the nonlinear spectral profiles across the evaluated purines. Furthermore, the complete set of PCA-based 2PA spectral reconstructions, alongside their respective RMSE evaluations, is detailed in **Figure SI6.**

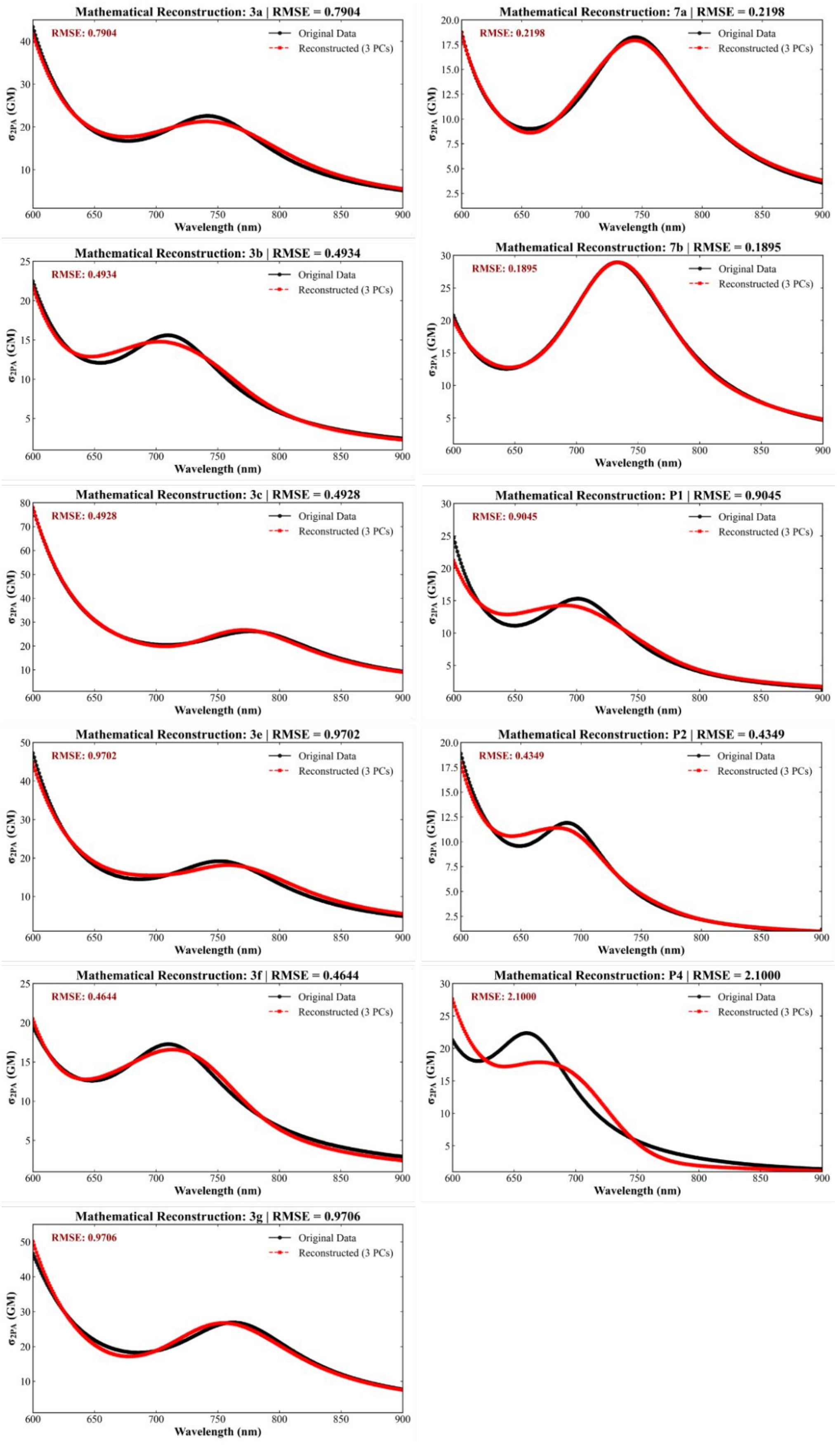

Figure S6. Reconstruction for all original spectra used in PCA of 2PA. The reconstructed spectra of 3d and P3 are presented and discussed in the main manuscript.
